# Worldwide burden of cancer attributable to diabetes and high body-mass index: a comparative risk assessment

**DOI:** 10.1016/S2213-8587(18)30150-5

**Published:** 2018-06

**Authors:** Jonathan Pearson-Stuttard, Bin Zhou, Vasilis Kontis, James Bentham, Marc J Gunter, Majid Ezzati

**Affiliations:** a School of Public Health, MRC-PHE Centre for Environment and Health, Imperial College London, London, UK; b Department of Epidemiology and Biostatistics, School of Public Health, Imperial College London, London, UK; c WHO Collaborating Centre on NCD Surveillance and Epidemiology, Imperial College London, London, UK; d School of Mathematics, Statistics and Actuarial Science (SMSAS), University of Kent, Canterbury, UK; e Nutrition and Metabolism Section, International Agency for Research on Cancer, World Health Organization, Lyon, France

## Abstract

**Background:**

Diabetes and high body-mass index (BMI) are associated with increased risk of several cancers, and are increasing in prevalence in most countries. We estimated the cancer incidence attributable to diabetes and high BMI as individual risk factors and in combination, by country and sex.

**Methods:**

We estimated population attributable fractions for 12 cancers by age and sex for 175 countries in 2012. We defined high BMI as a BMI greater than or equal to 25 kg/m^2^. We used comprehensive prevalence estimates of diabetes and BMI categories in 2002, assuming a 10-year lag between exposure to diabetes or high BMI and incidence of cancer, combined with relative risks from published estimates, to quantify contribution of diabetes and high BMI to site-specific cancers, individually and combined as independent risk factors and in a conservative scenario in which we assumed full overlap of risk of diabetes and high BMI. We then used GLOBOCAN cancer incidence data to estimate the number of cancer cases attributable to the two risk factors. We also estimated the number of cancer cases in 2012 that were attributable to increases in the prevalence of diabetes and high BMI from 1980 to 2002. All analyses were done at individual country level and grouped by region for reporting.

**Findings:**

We estimated that 5·7% of all incident cancers in 2012 were attributable to the combined effects of diabetes and high BMI as independent risk factors, corresponding to 804 100 new cases. 187 600 (24·5%) of 766 000 cases of liver cancer and 121 700 (38·4%) of 317 000 cases of endometrial cancer were attributable to these risk factors. In the conservative scenario, about 4·5% (629 000 new cases) of all incident cancers assessed were attributable to diabetes and high BMI combined. Individually, high BMI (544 300 cases) was responsible for almost twice as many cancer cases as diabetes (293 300 cases). 25·8% of diabetes-related cancers (equating to 75 600 new cases) and 31·9% of high BMI-related cancers (174 040 new cases) were attributable to increases in the prevalence of these risk factors from 1980 to 2002.

**Interpretation:**

A substantial number of cancer cases are attributable to diabetes and high BMI. As the prevalence of these cancer risk factors increases, clinical and public health efforts should focus on identifying optimal preventive and screening measures for whole populations and individual patients.

**Funding:**

NIHR and Wellcome Trust.

## Introduction

Diabetes and high body-mass index (BMI), defined as a BMI greater than or equal to 25 kg/m^2^, are leading causes of mortality and morbidity globally^1^ and their prevalence has increased substantially over the past four decades in most countries.^2^, ^3^ The global age-standardised adult prevalence of diabetes was reported to be 9·0% in men and 7·9% in women in 2014, affecting about 422 million adults.^3^ In 2016, the age-standardised adult prevalence of overweight and obesity (those with BMI ≥25 kg/m^2^) was estimated to be 38·5% in men and 39·2% in women, affecting approximately 2·01 billion adults globally.^2^

The International Agency for Research on Cancer (IARC) and the World Cancer Research Fund (WCRF) have concluded that there is a causal association between high BMI and colorectal,^4^ gallbladder,^5^ pancreas,^6^ kidney,^7^ liver,^8^ endometrial,^9^ postmenopausal breast,^10^ ovarian,^11^ gastric cardia,^12^ and thyroid cancer,^13^ as well as oesophageal adenocarcinoma^14^ and multiple myeloma.^13^ A study in 2015 estimated that about 3·6% of all cancer cases in 2012 were attributable to high BMI.^15^ Since then, high BMI has been thought to have a causal relationship with additional site-specific cancers^8^, ^13^, ^14^, ^16^ and more recent and more detailed global BMI prevalence estimates, based on substantially more data, have become available.^2^ Diabetes is increasingly recognised as a risk factor for colorectal, pancreatic, liver, gallbladder, breast, and endometrial cancer,^17^ but the global cancer burden attributable to diabetes has not been quantified. Furthermore, since high BMI is an important risk factor for diabetes, priority setting for public health and clinical interventions requires information on the cancer burden attributable to both high BMI and diabetes. We aimed to estimate the proportion of global cancer incidence in 2012 that was attributable to diabetes and high BMI individually and combined, under varying assumptions about the independence of their effects.

**Research in context**

**Evidence before this study**

We searched MEDLINE via PubMed for articles published up to June 30, 2017, with no language restrictions using the search terms (“Diabetes” OR “Body-mass index” OR “Overweight”, OR “Obesity”), AND (“Cancer risk”, OR “Cancer incidence”), AND “Attributable fraction”. We found one study estimating the burden of cancer associated with type 2 diabetes in 2010 and 2030 in Japan and we found several studies estimating the burden of cancer attributable to high BMI or obesity alone, either in one country or in one country and one cancer site. One previous study quantified the global burden of cancer attributable to high BMI. New, more comprehensive estimates of BMI prevalence have since been published. No previous study has estimated the global burden of cancer attributable to diabetes alone or diabetes and high BMI combined.

**Added value of this study**

To our knowledge, this study provides the first estimate of global cancer burden attributable to diabetes alone and to diabetes and high BMI combined, and uses the most comprehensive available estimates of diabetes and high BMI prevalence. We also quantified the global burden of cancer attributable to rises in the prevalence of diabetes and high BMI over time.

**Implications of all the available evidence**

In 2012, about 6% of all incident cancers were attributable to the combined effects of diabetes and high BMI, corresponding to 804 100 cases. As the prevalence of these cancer risk factors increases, clinical and public health efforts should focus on identifying optimal preventive and screening measures for whole populations and individual patients.

## Methods

### Study design

We reviewed the WCRF continuous update projects, IARC publications, and other published literature that summarised associations of diabetes^17^ and high BMI with site-specific cancers.^4^, ^5^, ^6^, ^7^, ^8^, ^9^, ^10^, ^11^, ^12^, ^13^, ^14^ We searched MEDLINE via PubMed for articles published up to June 30, 2017, with no language restrictions using the search terms (“Diabetes” OR “Body-mass index” OR “Overweight”, OR “Obesity”), AND (“Cancer risk”, OR “Cancer incidence”), AND “Attributable fraction”. We selected cancers that the WCRF and IARC have judged to have a causal association with high BMI: colorectal, gallbladder, pancreatic, liver, postmenopausal breast, endometrial, kidney, ovarian, stomach cardia, and thyroid cancer, oesophageal adenocarcinoma, and multiple myeloma. For diabetes, we identified published meta-analyses^17^ of the relative risks (RR) for the association of diabetes with site-specific cancer. The studies included in the meta-analyses had applied rigorous adjustment to control for potential confounding factors, including BMI. The RRs for each site-specific cancer applied in our analysis and their sources are detailed in the appendix 2 (pp 1, 2). For the diabetes analysis we included colorectal, gallbladder, pancreatic, liver, breast, and endometrial cancer.

High BMI has also been proposed to be causally associated with meningioma.^13^ However, most meningiomas are benign and the incidence of meningioma is not reported in GLOBOCAN. The association between high BMI and oesophageal and stomach cancer is limited to oesophageal adenocarcinoma^14^ and stomach cardia^12^ cancer; therefore, we only included these two subtypes in our analysis.

Using prevalence of diabetes^3^ and of categories of BMI^2^ and RRs for their associations with the cancers identified from published meta-analyses, we estimated the population attributable fraction (PAF) of incident cancers attributable to diabetes and high BMI. For 175 countries in 2012 (appendix 2 p 10), we estimated individual PAFs for each risk factor, as well as two scenarios of diabetes and high BMI combined, one treating their effects as independent and another as overlapping. All analyses were stratified by sex and age group and restricted to people aged 18 years or older. We then estimated the number of cancer cases attributable to diabetes, high BMI, and their combined effect globally by multiplying the PAFs with the number of incident cancers for each age, sex, and country stratum using data from GLOBOCAN.^18^

Given the cumulative nature of carcinogenesis, and the importance of risk factor exposure over time, a time lag of several years from exposure to the risk factor and development of the disease is expected. For the association between high BMI and cancer, this lag is commonly assumed to be about 10 years.^19^ Thus, in our main analysis we calculated cancer incidence in 2012 that we attributed to diabetes and high BMI in 2002. We also estimated cancer incidence due to the change in the prevalence of these two risk factors from 1980 to 2002.

### Data sources

We obtained data on the prevalence of diabetes and categories of BMI for 1980 and 2002, stratified by age group (18–19, 20–24, 25–29, 30–34, 35–39, 40–44, 45–49, 50–54, 55–59, 60–64, 65–69, 70–74, 75–79, 80–84, and ≥85 years), sex, and country from estimates^2^, ^3^ by the NCD Risk Factor Collaboration (NCD-RisC). BMI data were summarised as prevalence of BMI categories (<18·5, 18·5 to <20, 20 to <25, 25 to <30, 30 to <35, 35 to <40, and ≥40 kg/m^2^) to characterise the varying shape of the distribution across populations.^2^ Diabetes was defined as fasting plasma glucose greater than or equal to 7·0 mmol/L, a history of diagnosis of diabetes (we did not differentiate between type 1 and type 2 diabetes), or use of insulin or oral hypoglycaemic drugs. The data sources used by NCD-RisC to estimate BMI and diabetes were checked against a defined set of inclusion criteria, which have been described in detail previously,^1^, ^2^ and data were reanalysed according to a common protocol. To avoid potential bias from self-reported data, NCD-RisC only uses data from studies that had measured height and weight or a diabetes biomarker (fasting plasma glucose, 2 h oral glucose tolerance test, or HbA_1c_). The same criteria and protocol were applied to studies throughout time and across countries. After pooling the data, NCD-RisC fitted a bespoke Bayesian hierarchical model to the data with the Markov chain Monte Carlo algorithm and generated 1000 draws from the posterior distribution for each country-year-sex-age stratum. Details have been reported previously in studies investigating BMI and diabetes.^2^, ^3^

GLOBOCAN 2012 cancer incidence data^18^ for the selected cancer sites were available in age groups (15–39, 40–44, 45–49, 50–54, 55–59, 60–64, 65–69, 70–74, and ≥75 years). We used population weighting to ensure that the age groups for diabetes and BMI prevalence were the same as those for cancer incidence. The GLOBOCAN cancer incidence data covered 183 countries and territories, for which both diabetes and BMI estimates were available in 175 of them. We subsequently grouped these 175 countries into nine regions by geographical and national income criteria (appendix 2 p 10).

### Statistical analysis

Most risk factors act proportionally to increase disease risk, therefore we first calculated the proportional reduction of cancer that would occur if exposure to the risk factor was reduced to an alternative scenario, as measured by the PAF.^20^ The PAF attributable to diabetes and high BMI separately was calculated using the formula^21^

$$PAF=\frac{\sum P_{i}RR_{i}-\sum P{'}_{i}RR_{i}}{\sum P_{i}RR_{i}}$$

where *P*_i_ is the actual prevalence of diabetes or BMI category *i, P*′_i_ is the prevalence in an alternative scenario, and *RR*_i_ the adjusted relative risk of site-specific cancer associated with diabetes or the corresponding level of BMI. In our main analysis we estimated the total cancer burden of diabetes and high BMI, and used an optimal prevalence as our alternative scenario—namely zero diabetes prevalence and BMI of 20–25 kg/m^2^ (used as 22·5 kg/m^2^ in the calculation), where the cancer risk is assumed to be lowest at the population level. A diabetes prevalence of less than 1% has not been observed,^22^ so we did a further analysis in which the optimal prevalence of diabetes was 1% rather than zero. We calculated PAFs for 2035 with prevalence in 2025 (projected on the assumption that recent trends continue, as described previously) instead of 2002 prevalence.^2^, ^3^, ^23^

Diabetes and high BMI have increased in prevalence substantially worldwide since 1980.^2^, ^3^ We therefore used a second alternative scenario to estimate the cancer burden attributable to these increases. To do this, we replaced the optimal prevalence with the prevalence of diabetes and high BMI in 1980 as the alternative scenario.

We then calculated the PAFs for the combined effects of diabetes and high BMI in two scenarios: diabetes and high BMI as independent risk factors, and a conservative estimate. To calculate combined PAF with high BMI and diabetes as independent risk factors, we used the formula^24^ PAF =        1 – [(1 – PAF_Diabetes_) × (1 – PAF_High BMI_)]. For the conservative estimate, we selected the larger of PAF_Diabetes_ and PAF_High BMI_ in each age, sex, and country stratum to generate a conservative PAF. This approach assumes complete overlap of pathophysiology of diabetes and high BMI with cancer.

We calculated the number of incident cancer cases in 2012 attributable to each risk factor individually and combined as the product of the corresponding PAF and the incident site-specific cancer cases. All analyses were done by sex, age group, and country stratum. To produce aggregated results across age groups, we weighted the age group-specific PAFs by age group-specific cancer incidence by sex and country.

We propagated the uncertainties of diabetes and BMI prevalence estimates and those of the RRs to the final estimates using a simulation approach. Specifically, we generated 1000 draws for each RR from a log-normal distribution, with mean equal to the reported estimate and SD calculated with the reported confidence interval and 1000 draws from the posterior distributions of diabetes^3^ and high BMI prevalence.^2^ We repeated the PAF calculation for each of these draws, resulting in 1000 PAFs which characterised the uncertainty distribution of the output. We report 95% uncertainty intervals (95% UI) for our estimates as the 2·5th to 97·5th percentile of the resultant distributions. All analyses were done with *R* version 3.2.5.^25^

### Role of the funding source

The funder of the study had no role in study design, data collection, data analysis, data interpretation, or writing of the report. JP-S, BZ, VK, and JB, had full access to all the data in the study and the corresponding author had final responsibility for the decision to submit for publication.

## Results

**Figure 1:**
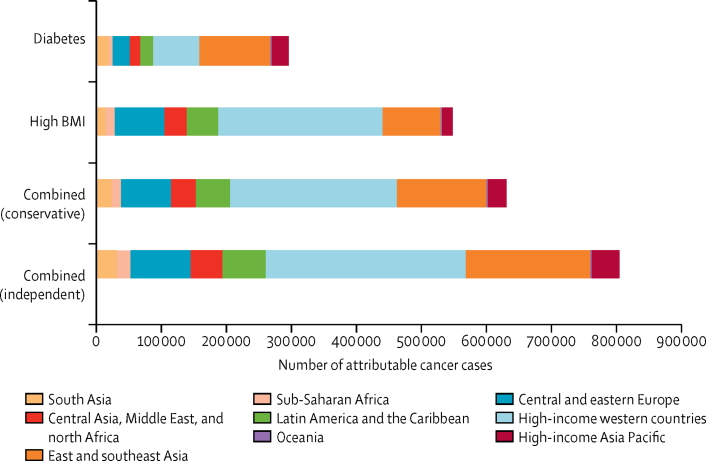
Global cancer cases in 2012 attributable to diabetes and high BMI, individually and combined, in the conservative and independent scenarios, by region BMI=body-mass index.

**Figure 2:**
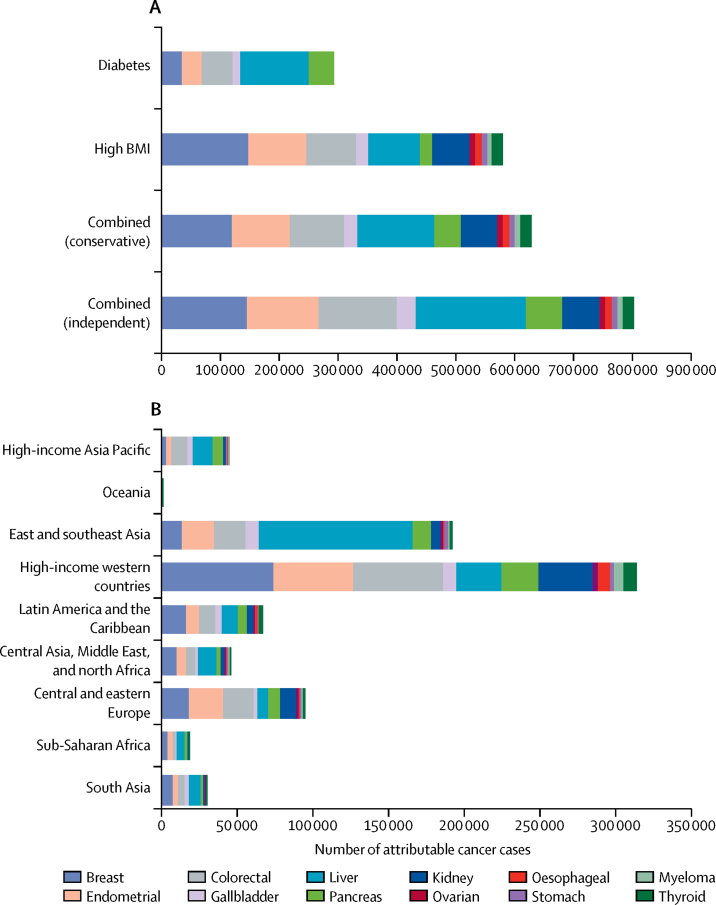
Global site-specific cancer cases in 2012 Cases by (A) diabetes and high BMI, individually and in combination, in the conservative and independent scenarios and (B) region, in the combined independent scenario. BMI=body-mass index.

In 2012, diabetes and high BMI combined were responsible for an estimated 804 100 new cases of cancer worldwide (5·7% of all 14 067 894 cancer cases reported by GLOBOCAN^18^) in the independent scenario. 293 300 (2·1%) cancer cases were attributable to diabetes and 544 300 (3·9%) to high BMI alone (Figure 1, Figure 2). In the conservative scenario, the two risk factors combined were responsible for 629 000 new cancer cases in 2012. Cancer cases attributable to diabetes and high BMI combined were almost twice as common in women (501 600 cases) as in men (302 500 cases) in the independent scenario.

In men, 126 700 cases (95% UI 95 900–159 400) were from liver cancer, constituting 41·9% of all cancer cases attributable to diabetes and high BMI combined in the independent scenario; colorectal cancer cases (69 800 cases, 56 200–83 700) were the next largest contributor, constituting 23·1% of the total cases (Figure 1, Figure 2; table 1). In women, there were 147 400 cases (106 700–190 000) of breast cancer, constituting 29·4% of all cancers attributable to diabetes and high BMI; the second largest contributor was endometrial cancer (121 700 cases, 108 600–135 000), which constituted 24·3% of such cases.

**Table 1:** PAF and number of cancer cases attributable to high BMI and diabetes in 2012, individually and in combination, in independent and conservative scenarios

	**Total number of cases**	**High BMI PAF**	**High BMI cases**	**Diabetes PAF**	**Diabetes cases**	**Independent PAF**	**Independent scenario cases**	**Conservative PAF**	**Conservative scenario cases**
**Men**									
Colorectal	736 000	5·8% (4·2–7·4)	42 200 (30 600–54 800)	4·0% (2·9–5·1)	29 000 (21 500–37 600)	9·5% (7·6–11·4)	69 800 (56 200–83 700)	6·5% (5·2–8·0)	48 000 (38 300–59 000)
Gallbladder	76 000	9·7% (5·8–13·2)	7400 (4500–10 100)	7·8% (4·0–11·9)	5900 (3000–9200)	16·7% (11·9–21·8)	12 800 (9100–16 600)	11·7% (8·2–15·4)	9000 (6300–11 800)
Liver	543 000	10·1% (5·7–14·7)	54 600 (31 100–79 600)	14·5% (0·8–19·7)	80 200 (54 700–107 800)	23·3% (17·6–29·3)	126 700 (95 900–159 400)	16·5% (12·4–21·2)	89 500 (67 600–115 400)
Pancreas	177 000	5·8% (3·9–7·8)	10 300 (6800–13 700)	12·8% (9·3–16·8)	22 700 (16 200–29 500)	18·0% (14·0–21·6)	31 900 (24 700–38 100)	13·2% (9·7–16·6)	23 300 (17 200–29 300)
Kidney	208 000	18·0% (15·5–20·4)	37 400 (32 100–42 300)	..	..	18·0% (15·5–20·4)	37 400 (32 100–42 300)	18·0% (15·5–20·4)	37 400 (32 100–42 300)
Oesophagus (adenocarcinoma)	31 700	28·7% (22·6–35·0)	9100 (7200–11 100)	..	..	28·7% (22·6–35·0)	9100 (7200–11 100)	28·7% (22·6–35·0)	9100 (7200–11 100)
Stomach (cardia)	72 700	8·8% (3·0–14·8)	6400 (2200–10 800)	..	..	8·8% (3·0–14·8)	6400 (2200–10 800)	8·8% (3·0–14·8)	6400 (2200–10 800)
Multiple myeloma	61 900	7·2% (3·3–11·1)	4500 (2100–6900)	..	..	7·2% (3·3–11·1)	4500 (2100–6900)	7·2% (3·3–11·1)	4500 (2100–6900)
Thyroid	67 000	5·8% (2·8–8·8)	3900 (1900–5900)	..	..	5·8% (2·8–8·8)	3900 (1900–5900)	5·8% (2·8–8·8)	3900 (1900–5900)
Total	1 973 300	8·9%	175 800	9·0%	137 800	15·3%	302 500	11·7%	231 100
**Women**									
Breast	1 656 000	6·9% (4·4–9·4)	114 800 (72 700–156 500)	2·2% (1·3–3·2)	36 200 (21 400–51 600)	8·9% (6·4–11·5)	147 400 (106 700–190 000)	7·2% (4·9–9·8)	120 000 (82 500–161 500)
Endometrial	317 000	31·0% (27·1–35·2)	98 400 (86 000–111 500)	10·8% (7·8–13·8)	33 700 (25 100–43 900)	38·4% (34·3–42·6)	121 700 (108 600–135 000)	31·3% (27·4–35·4)	99 100 (87 000–112 200)
Colorectal	607 000	7·0% (5·0–9·1)	42 300 (30 200–55 000)	3·8% (2·8–4·8)	22 700 (16 600–29 200)	10·5% (8·5–12·6)	63 500 (51 800–76 800)	7·3% (5·8–9·2)	44 600 (34 900–55 900)
Gallbladder	101 000	12·9% (7·8–17·6)	13 000 (7900–17 700)	7·4% (4·0–11·5)	7600 (3800–11 500)	19·3% (13·6–25·1)	19 400 (13 700–25 200)	13·8% (9·4–18·1)	13 900 (9500–18 300)
Liver	223 000	13·5% (7·8–19·4)	30 200 (17 400–43 200)	15·8% (10·9–21·4)	35 300 (24 400–47 200)	27·3% (20·9–33·9)	60 900 (46 500–75 600)	18·8% (14·4–23·8)	42 000 (32 100–53 000)
Pancreas	159 000	7·1% (4·6–9·4)	11 200 (7300–15 000)	12·6% (9·2–16·6)	20 000 (14 500–26 200)	19·0% (14·6–22·7)	30 100 (23 200–36 100)	13·1% (9·8–16·5)	20 700 (15 600–26 300)
Kidney	118 000	21·3% (18·3–24·1)	25 200 (21 600–28 500)	..	..	21·3% (18·3–24·1)	25 200 (21 600–28 500)	21·3% (18·3–24·1)	25 200 (21 600–28 500)
Ovarian	235 000	3·9% (0·9–6·7)	9100 (2000–15 800)	..	..	3·9% (0·9–6·7)	9100 (2000–15 800)	3·9% (0·9–6·7)	9100 (2000–15 800)
Oesophagus (adenocarcinoma)	7300	29·5% (23·1–36·1)	2200 (1700–2600)	..	..	29·5% (23·1–36·1)	2200 (1700–2600)	29·5% (23·1–36·1)	2200 (1700–2600)
Stomach (cardia)	26 400	11·2% (3·8–18·8)	2900 (1000–5000)	..	..	11·2% (3·8–18·8)	2900 (1000–5000)	11·2% (3·8–18·8)	2900 (1000–5000)
Multiple myeloma	51 400	8·9% (4·0–13·3)	4400 (2000–6800)	..	..	8·9% (4·0–13·3)	4400 (2000–6800)	8·9% (4·0–13·3)	4400 (2000–6800)
Thyroid	226 400	6·5% (3·2–9·8)	14 800 (7300–22 100)	..	..	6·5% (3·2–9·8)	14 800 (7300–22 100)	6·5% (3·2–9·8)	14 800 (7300–22 100)
Total	3 727 500	9·9%	368 500	5·1%	155 500	13·5%	501 600	10·7%	397 900

Numbers in parentheses show 95% UI. PAF=population attributable fraction. BMI=body-mass index.

Of the six cancers associated with diabetes and 12 associated with high BMI, 15·3% in men and 13·5% in women were attributable to the combined effect of these risk factors in the independent scenario (11·7% in men and 10·7% in women in the conservative scenario; table 1). The PAF varied substantially by cancer site in both sexes. Of all liver cancers, 23·3% (17·6–29·3) in men and 27·3% (20·9–33·9) in women were attributable to diabetes and high BMI combined, compared with just 9·5% (7·6–11·4) of cases of colorectal cancer in men and 10·5% (8·5–12·6) in women. 38·4% (34·3–42·6) of all endometrial cancer cases in 2012 were attributable to these risk factors compared with 3·9% (0·9–6·7) of ovarian cancer cases (table 1).

There were notable differences in the proportion of cancer cases attributable to diabetes versus high BMI individually. For example, high BMI was responsible for about three times the proportion of breast (6·9%) and endometrial (31·0%) cancers as compared with diabetes (2·2% for breast and 10·8% for endometrial; table 1). By contrast, the proportion of liver (14·5%) and pancreatic (12·8%) cancer in men attributable to diabetes was substantially larger than that attributable to high BMI (10·1% for liver and 5·8% for pancreatic). When using 1% as the optimal diabetes prevalence rather than zero, this resulted in a reduction in cancer cases attributable to diabetes by 6·6% (274 000 *vs* 293 300).

313 000 (38·9%) of 804 100 cases of cancer attributable to the combined risk of diabetes and high BMI in the independent scenario in 2012 occurred in high-income western countries (Figure 1, Figure 2). East and southeast Asia had the second largest proportion (191 900 [23·8%]) of cases attributable to the combined risk of diabetes and high BMI, and the largest number of cancer cases attributable to diabetes individually (108 700 attributable cases) (figure 2).

The contribution of each cancer site to the regional cancer burden also varied substantially. Of the total cancer burden due to the combination of diabetes and high BMI, liver cancer contributed more than 29·6% in the high-income Asia Pacific region and 53·1% in east and southeast Asia, compared with just 6·9% in central and eastern Europe (figure 2B). By contrast, breast and endometrial cancer contributed about 18·3% of the combined cancer burden in east and southeast Asia and 15·1% in the high-income Asia Pacific region, compared with roughly 40·5% in high-income western countries, central and eastern Europe, and sub-Saharan Africa. There were substantial differences in the PAF of cancer attributable to diabetes and those attributable to high BMI in some regions, for example in women in central Asia, the Middle East, and north Africa (5·8% for diabetes *vs* 14·3% for high BMI; table 2), and in men in east and southeast Asia (10·3% for diabetes *vs* 5·6% for high BMI)—where diabetes^3^ has increased faster than expected by the rise in BMI.^2^

**Table 2:** Regional cancer cases in 2012 attributable to 2002 prevalence and cancer cases that would have been expected in 2012 had prevalence remained at 1980 levels

		**Number of cases**	**Cases attributable to 2002 prevalence**	**Proportion of cases attributable to 2002 prevalence**	**Cases attributable to 1980 prevalence**	**Proportion of cases attributable to 1980 prevalence**
**Diabetes**						
Men						
	Central and eastern Europe	114 000	7400 (5800–9300)	6·5%	6000 (3800–9900)	5·3%
	Central Asia and north Africa and the Middle East	56 000	6500 (4900–8300)	11·6%	4100 (2100–6900)	7·3%
	East and southeast Asia	616 000	63 600 (44 100–84 400)	10·3%	37 500 (15 300–73 800)	6·1%
	High-income Asia Pacific region	157 000	15 000 (11 300–19 300)	9·6%	11 800 (7200–17 400)	7·5%
	High-income western countries	385 000	28 700 (20 700-37 200)	7·5%	22 500 (14 200–35 200)	5·8%
	Latin America and the Caribbean	76 000	6700 (4900–8600)	8·8%	5100 (3100–8100)	6·7%
	Oceania	800	90 (60–120)	11·3%	50 (20–100)	6·3%
	South Asia	83 000	7100 (5400–8800)	8·6%	3700 (1200–6700)	4·5%
	Sub-Saharan Africa	44 000	3000 (2200–4100)	6·8%	1700 (700–3500)	3·9%
Women						
	Central and eastern Europe	297 000	16 800 (13 200–20 800)	5·7%	15 800 (9600–24 000)	5·3%
	Central Asia and north Africa and the Middle East	149 000	8600 (6900–10 400)	5·8%	5500 (3000–9400)	3·7%
	East and southeast Asia	720 000	45 100 (34 000–57 300)	6·3%	35 000 (16 500–64 700)	4·9%
	High-income Asia Pacific region	201 000	12 100 (9500–15 400)	6·0%	10 900 (7000–15 800)	5·4%
	High-income western countries	1 019 000	43 200 (34 100–53 900)	4·2%	38 000 (25 500–56 600)	3·7%
	Latin America and the Caribbean	254 000	13 900 (11 000–17 600)	5·5%	10 700 (6300–16 900)	4·2%
	Oceania	2000	130 (90–180)	6·5%	70 (30–150)	3·5%
	South Asia	283 000	11 100 (8400–14 400)	3·9%	6600 (3000–13 500)	2·3%
	Sub-Saharan Africa	138 000	4500 (3400–5800)	3·3%	2900 (1400–5700)	2·1%
**High BMI**						
Men						
	Central and eastern Europe	146 000	18 800 (15 100–22 700)	12·9%	13 400 (10 400–16 900)	9·2%
	Central Asia and north Africa and the Middle East	67 000	9800 (7200–12 600)	14·6%	6100 (4200–8400)	9·1%
	East and southeast Asia	711 000	40 000 (25 800–56 100)	5·6%	16 500 (9500–23 500)	2·3%
	High-income Asia Pacific region	182 000	8600 (6300–11 100)	4·7%	4900 (3500–6800)	2·7%
	High-income Western countries	502 000	82 200 (65 200–99 000)	16·4%	57 900 (44 900–70 900)	11·5%
	Latin America and the Caribbean	94 000	12 300 (9600–15 000)	13·1%	7300 (5500–9600)	7·8%
	Oceania	800	100 (60–130)	12·5%	60 (40–90)	7·5%
	South Asia	96 000	2600 (1900–3500)	2·7%	1100 (600–1700)	1·1%
	Sub-Saharan Africa	46 000	2000 (1300–2800)	4·3%	900 (600–1500)	2·0%
Women						
	Central and eastern Europe	348 000	58 700 (49 100–68 500)	16·9%	51 700 (42 600–61 400)	14·9%
	Central Asia and north Africa and the Middle East	167 000	23 800 (19 100–28 400)	14·3%	16 800 (12 900–21 000)	10·1%
	East and southeast Asia	815 000	48 000 (38 400–57 700)	5·9%	25 100 (18 500–33 300)	3·1%
	High-income Asia Pacific region	224 000	10 900 (8600–13 400)	4·9%	8600 (6600–10 800)	3·8%
	High-income western countries	1 136 000	170 200 (138 000–202 300)	15·0%	124 200 (100 000–149 600)	10·9%
	Latin America and the Caribbean	281 000	37 700 (30 500–45 000)	13·4%	26 600 (21 000–32 900)	9·5%
	Oceania	2000	300 (230–370)	15·0%	200 (140–270)	10·0%
	South Asia	323 000	9800 (7400–12 300)	3·0%	4700 (3000–6700)	1·5%
	Sub-Saharan Africa	153 000	9700 (7700–11 800)	6·3%	5400 (4100–7000)	3·5%

Data are stratified by sex. Numbers in parentheses are 95% UI. BMI=body-mass index.

**Figure 3:**
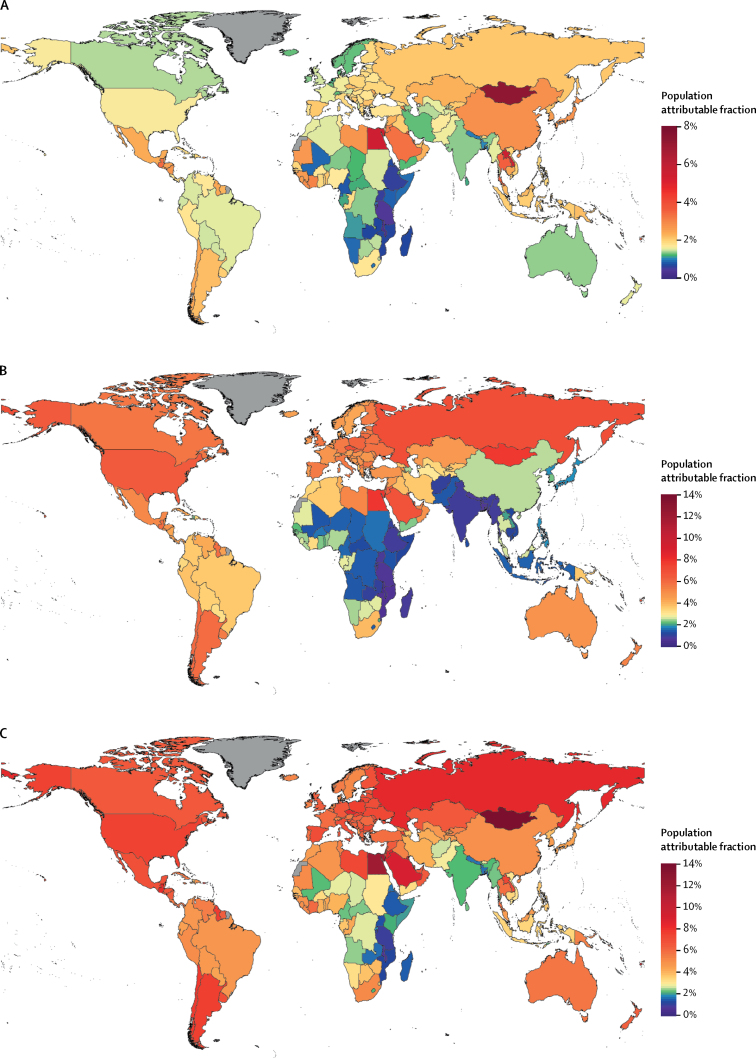
Population attributable fraction of all cancer incidence in 2012 Population attributable fractions shown are those of (A) diabetes, (B) high BMI, and (C) diabetes and high BMI combined as independent risks. Countries shown in grey did not have cancer incidence data. BMI=body-mass index.

There was substantial heterogeneity in the proportion of cancer cases attributable to diabetes, high BMI, and their combination in the independent scenario at country level. For example, less than 1% of all new cancer cases in Malawi (0·6%) and Tanzania (0·9%) in 2012 were attributable to diabetes and high BMI combined, compared with more than 10% in Egypt (12·0%) and Mongolia (13·9%)—the countries with the largest PAF—reflecting large variations in risk factor prevalence, and in the way that some cancers are more affected by these factors than others (figure 3).

We calculated that 25·8% of all cancer cases in 2012 attributable to diabetes were due to the increase in diabetes prevalence from 1980 to 2002 (table 2), equating to 75 600 new cases worldwide. 31·9% of cancer cases attributable to high BMI were due to increased prevalence of this risk factor over the same period, accounting for approximately 174 040 cancer cases. The largest proportion of cancer cases attributable to the increase in prevalence of diabetes and high BMI during this period was in low-income and middle-income countries (LMICs) in Asia and sub-Saharan Africa. At the two extremes, just 3% of cancer cases attributable to diabetes were due to increased diabetes prevalence in women in central and eastern Europe, compared with 57·2% in men in east and southeast Asia.

The PAF of cancer attributable to diabetes and high BMI is expected to increase substantially in coming decades (appendix 2 p 5). For example, PAFs for most site-specific cancers would increase by more than 30% in women and 20% in men when using projected 2025 prevalence compared with 2002 prevalence. In men, the PAF for liver cancer would increase by 47% (from 23·3% to 34·3%) and gallbladder cancer would increase by 53% (from 16·7% to 25·5%), while in women, the PAF for ovarian cancer would increase by 38% (from 3·9% to 5·4%).

## Discussion

We estimated that approximately 6% of cancer cases worldwide in 2012 were attributable to diabetes and high BMI, with high BMI being responsible for almost twice as many cases as diabetes. About a third of cancer cases attributable to diabetes and a quarter of cases attributable to high BMI were due to increases in the prevalence of these risk factors from 1980 to 2002. Given the continued rise in the prevalence of these risk factors since 2002,^2^, ^3^ the attributable cancer burden is likely to continue to increase in coming decades. Approximately one in four liver and oesophageal adenocarcinomas and 38·4% of endometrial cancers worldwide in 2012 were estimated to be attributable to diabetes and high BMI.

LMICs have had substantial increases in the prevalence of diabetes and high BMI during the past three decades, whereas parts of Europe and the high-income Asia Pacific region have seen more stable age-standardised prevalences (appendix 2 p 7).^2^, ^3^ In our analysis LMICs had the largest increases in numbers of cancer cases attributable both to diabetes, and diabetes and high BMI combined, which is particularly important to note because these countries are generally less well equipped to manage the burden of complex non-communicable diseases (NCDs) than high-income countries.

Previous studies have quantified the global cancer burden attributable to nine potentially modifiable diet and lifestyle risk factors (PAF 35% in 2001),^26^ smoking (PAF 21% in 2000),^27^ high BMI (PAF 3·6% in 2012),^15^ and common infections (PAF 15·4% in 2012).^28^ Our findings suggest that 3·9% of global cancer cases in 2012 were attributable to high BMI, taking into account the four additional cancer sites and more comprehensive and up-to-date BMI data compared with previous work.^15^

Proposed biological mechanisms underlying the link between diabetes, high BMI, and cancer include hyperinsulinaemia, hyperglycaemia, chronic inflammation,^29^ and dysregulation of sex hormone activity. Insulin itself could be oncogenic,^30^ and results from several analyses showed that people with hyperinsulinaemia were at increased risk of breast and colorectal cancer irrespective of their BMI.^31^, ^32^, ^33^ Prospective studies and large-scale consortia with more accurate assessments of adiposity, diabetes, and metabolic health, which incorporate molecular tools, will be needed to draw conclusions about the underlying mechanisms that link diabetes, high BMI, and cancer, and inform clinical interventions.

To our knowledge, this is the only study to have quantified the global burden of cancer attributable to diabetes and to diabetes combined with high BMI, by use of robust evidence from WCRF^4^, ^5^, ^6^, ^7^, ^8^, ^9^, ^10^, ^11^, ^12^, ^14^ for BMI and high quality meta-analyses for diabetes.^17^ Our findings are important to policy makers developing coordinated approaches to tackle the rising prevalence of diabetes, high BMI, and all of their sequelae. The cancers judged to have a convincing association with diabetes by the umbrella meta-analysis were restricted to those for which the effect of study bias was expected to be lowest.

Our study has some limitations. The precision of the risk estimates used to adjust for common confounders, including diabetes and BMI, might be affected by potential biases such as reverse causality and ascertainment bias, which are believed to affect some estimates of the association between diabetes and cancer.^34^ We used the same relative risk for age group, sex, and region; more granular risk estimates by age, sex, and stage of diagnosis would allow for greater accuracy at the subgroup level. We quantified the cancer burden attributable to all BMI levels greater than 25 kg/m^2^. Some researchers have argued that Asian populations might need BMI cutoffs that are different from other populations,^35^ although meta-analyses of Asian and western cohorts have shown that disease risk increases by similar proportions in Asian and western populations^36^, ^37^, ^38^, ^39^ and indeed the latest WHO consensus statement on BMI cutoffs, having considered the arguments for region-specific cutoffs, recommended use of similar cutoffs throughout the world.^35^ The mediated and direct effects of diabetes and high BMI on cancer—which would allow for more accurate estimation of their combined contributions to the cancer burden—have not yet been quantified in the way that has been done for cardiovascular diseases.^40^ Additionally, the 10-year lag from diabetes and high BMI prevalence to cancer incidence that we used is an imperfect measure of cumulative past risk factor exposure, which is important for cancer burden.^41^ Our PAF analysis quantified the proportion and number of cancer cases that would be averted if diabetes and high BMI prevalence were reduced to optimal levels. However, if the cancer burden of diabetes and high BMI is removed, these risks could lead to populations developing other disorders such as cardiovascular disease and chronic kidney disease as quantified elsewhere.^42^ Finally, we assumed an optimal diabetes prevalence of zero, and achieving a prevalence of less than 1% might not be feasible.^22^ Nonetheless, when we substituted zero for 1% as the optimal diabetes prevalence, the cancer burden attributable to diabetes changed by less than 7% and was still responsible for 274 000 cases.

Trends in diabetes and those in BMI were only partly correlated across regions. For example, in south Asia and possibly east Asia diabetes prevalence has risen faster than would be expected by changes in BMI levels, whereas in northern Europe diabetes prevalence is increasing at a slower rate than might be expected by the changes in BMI. Several factors might be causing these diverse trends. First, regional differences in the prevalence of diabetes might be due to differences in genetic susceptibility or phenotypic variations arising from inadequate fetal and childhood nutrition and growth; earlier onset of β-cell dysfunction could be a differentiating characteristic of Asian populations compared with other groups.^43^, ^44^, ^45^, ^46^, ^47^ Second, people who are at high risk of developing diabetes might be identified at an earlier stage in health systems in high-income countries, allowing for earlier intervention with lifestyle and dietary modification or drugs.^48^ Finally, total caloric intake, dietary composition, and physical activity might affect diabetes risk and contribute to differences in regional trends to a greater extent than would otherwise be expected on the basis of BMI.^49^

Our results suggest that the increases in diabetes and BMI worldwide could lead to a substantial increase in the cancer burden in future decades. For example, when we used 2025 projections for diabetes and BMI prevalence we found that a substantially larger share of cancers would be attributable to these risk factors in the future than in 2012. PAFs for all site-specific cancers would be significantly higher if trends in diabetes and BMI continue as projected, with the largest increases in gallbladder, liver, and endometrial cancers. These projections are particularly alarming in view of the high, and growing, economic cost of cancers and metabolic diseases, and highlight the importance of integrated control measures to tackle common modifiable risk factors, alongside clinician awareness of diabetes and high BMI as established risk factors for common cancers.

Population-based strategies to prevent diabetes and high BMI have great potential impact—not least because many NCDs have overlapping risk factors, comorbidities, and shared sequelae—but have so far often failed, largely because of reluctance by governments and policy makers to pursue structural interventions that tackle key risks for NCDs, such as diet and physical inactivity.^1^ Future efforts should focus on identifying the most effective clinical interventions to prevent development of NCDs in at-risk groups and their sequelae, such as cancer. Primary care interventions, such as glucose-modifying medications, can be effective in preventing diabetes complications such as macrovascular disease,^50^ but this approach relies on early identification and close monitoring of people with diabetes, which can be challenging in LMICs that have limited resources. As well as coordinated approaches to halt and reverse the rise in NCDs, global efforts and clinical guidance should reflect the importance of cancer as a sequela of both diabetes and high BMI, and NCD control measures should be integrated into clinical guidelines to identify opportunities to reduce morbidity in this group of patients.

A previous version of this Article has been retracted, for changes made see appendix 1
